# Intelligence profiles and adaptive behaviors of high-functioning autism spectrum disorder and developmental speech and language disorders

**DOI:** 10.3389/fped.2022.972643

**Published:** 2023-01-09

**Authors:** Wen-Yuan Jin, Ling-Ling Wu, Li-Fei Hu, Wen-Hao Li, Chao Song, Yan-Yan Wang, Xiao-Lin Liu, Zhi-Wei Zhu

**Affiliations:** Department of Developmental Behavioral Pediatrics, Children's Hospital, Zhejiang University School of Medicine, National Clinical Research Center for Child Health, Hangzhou, China

**Keywords:** high-functioning autism spectrum disorder, developmental speech and language disorders, intelligence, adaptive behavior, WISC, ABAS

## Abstract

**Objective:**

The present study was aimed at investigating the intelligence profiles and adaptive behaviors of children with high-functioning autism spectrum disorder (HFASD) and developmental speech and language disorders (DSLDs). We compared the similarities and differences of cognitive capabilities and adaptive functions and explored their correlations in the HFASD and DSLDs groups.

**Methods:**

128 patients with HFASD, 111 patients with DSLDs and 114 typically developing (TD) children were enrolled into our study. Wechsler Intelligence Scale for Children-IV (WISC-IV) and Adaptive Behavior Assessment System-II (ABAS-II) were respectively applied to evaluate intelligence profiles and adaptive behaviors. Intelligence quotient (IQ) scores and adaptive functioning scores among the HFASD, DSLDs and TD groups were compared through one-way ANOVA. Pearson correlation coefficient was applied to examine the relationships between WISC indices and ABAS domains.

**Results:**

Outcomes showed significantly poorer intelligence profiles and adaptive behaviors in HFASD and DSLDs groups. Both children with HFASD and DSLDs demonstrated impairments in verbal comprehension and executive functions. Processing speed and working memory were the predominant defects of children with HFASD and DSLDs in the field of executive functions, respectively. Whereas perceptual reasoning was a relative strength for them. Children with DSLDs had balanced scores of all the domains in ABAS-II; nevertheless, HFASD individuals demonstrated striking impairments in Social domain. Correlation analysis showed IQs of children with HFASD were positively correlated with all the domains and General Adaptive Composite (GAC) of ABAS-II. Additionally, IQs were positively correlated with Conceptual domain and GAC for children with DSLDs. Compared with DSLDs group, intelligence displayed stronger correlations with adaptive behaviors in HFASD group.

**Conclusion:**

Our study expanded insights regarding intelligence profiles and adaptive behaviors of children with HFASD and DSLDs. Moreover, this study made breakthroughs in discovering positive correlations between IQs and adaptive functions in the two neurodevelopmental disorders.

## Introduction

Based on the Diagnostic and Statistical Manual of Mental Disorders, Fifth Edition (DSM-5), autism spectrum disorder (ASD) is a kind of neurodevelopmental disorder characterized by impaired social communications, restricted interests and repetitive patterns of behavior (1). According to the Autism and Developmental Disabilities Monitoring (ADDM) Network in United States, the prevalence of ASD is 23.0 per 1,000 (one in 44) children aged 8 years (2). About 31% ASD people are accompanied by intellectual disability (Full-Scale Intelligence Quotient, FSIQ <70) (1). Those ASD individuals without intellectual disability (FSIQ ≥70) are defined as high-functioning ASD (HFASD) (3). Intelligence profiles of HFASD have presented several specific characteristics. Evidence from previous studies suggested that subjects with HFASD showed impairments in Verbal Comprehension Index (VCI), Processing Speed Index (PSI) and FSIQ compared with typically developing (TD) children. PSI (Coding and Symbol Search) was always the lowest index, associated with increased autism communication symptoms (4, 5). In addition, Nader et al. (6) and Mayes et al. (7) discovered that HFASD people had defects in Working Memory Index (WMI), especially in Digit Span and Letter-Number Sequencing. Nevertheless, research findings indicated that HFASD individuals showed a good competence in Matrix Reasoning and Perceptual Reasoning Index (PRI) (4–8).

Developmental speech and language disorders (DSLDs) are defined as communication impairments that interfere the development of speech and language skills in the absence of cognitive disabilities, hearing loss, neurological or psychiatric disorders (9, 10). The prevalence of DSLDs ranges from 2.2% to 15%, covering multiple languages and cultures (11, 12). Subjects with DSLDs may suffer from speech disorders or language disorders, or both of them. DSLDs can affect children's abilities in language comprehension (receptive language) and verbal communication (expressive language). When children with DSLDs grew up, they had elevated risks of learning difficulties and poor performance on school achievements (13). It has been reported that DSLDs subjects had predisposition to dyslexia and problems in executive functions (14, 15). Additionally, increased evidence indicated that children with DSLDs were more prone to having difficulties in motor performance (9, 16). Therefore, DSLDs have distinctive defects in intellectual capabilities as a kind of neurodevelopmental disorder.

Several adaptive behavior measures, including Adaptive Behavior Assessment System (ABAS), Vineland Adaptive Behavior Scales (VABS) and Behavior Assessment System for Children (BASC), have been applied to evaluate the adaptive functions of children and adolescents with HFASD (17–20). All the scales suggested HFASD individuals had significant impairments on adaptive behaviors. Composite scores and all the skill area scores of HFASD group were generally more than one standard deviation (SD) below the population mean (17, 18). Social skill area was the most severe impaired part according to ABAS-Second Edition (ABAS-II). Daily living skill was another significant weakness of HFASD subjects based on VABS (18–20). Functional Academics was a relative strength of HFASD compared with other skill areas. Furthermore, results from previous studies revealed significant discrepancies between HFASD people's IQs and their adaptive functions (17, 18). Kenworthy et al. (20) discovered that communication skills were positively associated with IQs whereas global adaptive functions were negatively associated with autistic symptoms for children with HFASD.

Different from research concerning HFASD, few studies systemically explored the adaptive behaviors of children and adolescents with DSLDs. Self reports from 6-year-old children with DSLDs indicated the most important problem in their daily life was difficulties in academic achievements at school (15). Outcomes of a recent study revealed adolescents with DSLDs showed poorer school adjustment, less adaptive skills and more emotional problems (21). Young adults with DSLDs had lower levels of social self-efficacy and self confidence and higher levels of shyness (22). They were at increased risks of experiencing difficulties in friendships and community integration when they grew up (13). However, their prosocial behaviors, such as being kind, empathetic, sharing and helpful with others, were still within the normal range and stable over time (23).

As described above, previous research has summarized several defects in specific areas of cognitive and adaptive capabilities for children with HFASD and DSLDs. Our study was focused on comparing the similarities and differences of intelligence profiles and adaptive functions among the HFASD, DSLDs and TD groups and exploring the correlations between IQs and adaptive behaviors. Wechsler Intelligence Scale for Children-Fourth Edition (WISC-IV) and ABAS-II have been respectively applied to evaluate the IQ scores and adaptive behavior scores of our participants. The present study was aimed at providing more supports for clinicians to have a comprehensive understanding of intelligence and adaptive behavior characteristics of HFASD and DSLDs.

## Materials and methods

### Participants

Between January 2019 and January 2022, outpatients aged 6–16 years old visited the Department of Developmental Behavioral Pediatrics, Children's Hospital, Zhejiang University School of Medicine and met the inclusion criteria were enrolled in the present study. Our study was approved by the hospital's Clinical Research Ethics Committee (No. 2022-IRB-099) and the informed consent was obtained from parents or caregivers of every participant. HFASD was diagnosed based on DSM-5 criteria, results of Autism Behavior Checklist (ABC) and determined by two professionally qualified developmental behavioral pediatrics clinicians. Children with HFASD had an FSIQ met or exceeded 70 (according to WISC-IV). DSLDs were characterized by developmental delays in speech and language in the absence of mental or physical retardation, hearing loss, emotional disorder, or environmental deprivation (16). According to ICD-11, DSLDs group included developmental speech sound disorder, developmental speech fluency disorder and developmental language disorder. Children with DSLDs also had an FSIQ score ≥70 (WISC-IV). Participants of TD group were recruited among the outpatients without organic diseases or psychiatric disorders. Exclusion criteria of TD group consisted of ASD, DSLDs, attention deficit hyperactivity disorder, intellectual disorders and other neurodevelopmental diseases; inherited metabolic diseases; serious heart, liver and kidney dysfunctions; traumatic brain injuries and cerebral vascular diseases. A total of 128 patients with HFASD, 111 patients with DSLDs and 114 TD controls participated in our study. All the participants had no psychotropic medication records during the last three months.

### Autism behavior checklist

ABC is a widely applied screening tool of ASD introduced into China by Yang in 1993 (24). ABC is consisted of 57 items and five domains (sensory, relating, body concept, language and social self-help domains). It can be used for individuals aged from 18 months to 35 years old. Children and adolescents are highly suspected of ASD if the total scores of ABC meet or exceed 67.

### Wechsler intelligence scale for children-fourth edition

WISC-IV is an extensively used measure of intellectual ability, which was introduced into China in 2009 by Zhang (25). WISC-IV is consisted of four index scores: Verbal Comprehension Index (VCI), Perceptual Reasoning Index (PRI), Working Memory Index (WMI) and Processing Speed Index (PSI). It has 10 core subtests and 5 supplemental subtests. The VCI contains Vocabulary, Similarity, Comprehension, Information and Word Reasoning subtests; the PRI contains Block Design, Picture Concept, Matrix Reasoning and Picture Completion subtests; the WMI contains Digit Span, Letter-Number Sequencing and Arithmetic subtests; the PSI contains Coding, Symbol Search and Cancellation subtests. FSIQ is summarized from VCI, PRI, WMI and PSI. In our study, WISC-IV has been conducted for every participant by trained clinicians.

### Adaptive behavior assessment system-second edition

ABAS-II is a comprehensive, individualized and psychometrically adaptive behavior scale with good reliability and validity. The scale comprises a General Adaptive Composite (GAC) score, three domain scores (Conceptual, Social and Practical) and 10 skill area scores (Communication, Community Use, Functional Academics, Home Living, Health and Safety, Leisure, Self-Care, Self-Direction, Social, and Work) (26). The Conceptual domain contains Communication, Functional Academics, and Self-Direction; the Social domain contains Social and Leisure; the Practical domain contains Community Use, Home Living, Health and Safety, and Self-Care. Given that the majority of our participants did not have work experience and were under 17 years old, Work skills had not been included into the scale. All the parents or caregivers of participants were required to finish the questionnaire. Finally, 125 copies of ABAS-II in HFASD group, 109 copies in DSLDs group and 108 copies in TD group had been received in the study.

### Statistical analysis

Normally and non-normally distributed variables were respectively presented as mean ± SD and median (interquartile range, IQR) in our study. Rates [*N* (%)] of basic demographic characteristics between HFASD and TD group, between DSLDs and TD group were compared with chi-square test. Existing differences of VCI, PRI, WMI, PSI, FSIQ scores and all the subtests of WISC-IV among the HFASD, DSLDs and TD groups were compared through one-way analysis of variance (ANOVA). Comparisons of GAC scores, Conceptual domain scores, Social domain scores, Practical domain scores and all the skill area scores of ABAS-II among the three groups were conducted through one-way ANOVA. Pearson correlation coefficient was applied to examine the relationships between IQ scores (FSIQ, VCI, PRI, WMI, PSI) and adaptive behavior scores (GAC, Conceptual domain, Social domain and Practical domain). All the analyses were performed with IBM SPSS statistics 25.0 version (SPSS Inc, Chicago, III, USA). *P*-values <0.05 were defined as statistically significant.

## Results

### Basic demographic characteristics of subjects

Basic demographic characteristics of subjects were presented in Table 1. Median ages of the HFASD, DSLDs and TD groups were respectively 6.8, 6.9 and 8.2. Male participants in HFASD and DSLDs groups were respectively 88.3% and 87.4%; whereas male participants in TD group was 67.5%. Both HFASD and DSLDs were more likely to occur in boys than in girls (*P* < 0.01). Children living in town/country were more tended to suffer from DSLDs (*P* < 0.01). Paternal and maternal education backgrounds of DSLDs group were remarkably poorer than TD group (*P* < 0.01). Only 30.6% of fathers and 22.5% of mothers in DSLDs group received university or higher education. However, the proportions were approximately 50% in TD group. In DSLDs group, 32.4% of fathers and 26.1% of mothers had an education background of junior school or even lower, which were significantly greater than those in TD group (*P* < 0.01). However, paternal and maternal education status of HFASD group had no significant differences with TD group. Most of the subjects in all groups had no or only one sibling.

**Table 1:** Basic demographic characteristics of subjects.

Characteristics	HFASD group (*n* = 128)	DSLDs group (*n* = 111)	TD group (*n* = 114)
Age [median (IQR)]	6.8 (6.3–7.6)	6.9 (6.3–7.6)	8.2 (6.7–10.2)
Gender [*N* (%)]			
Male	113 (88.3%)	97 (87.4%)	77 (67.5%)
Female	15 (11.7%)	14 (12.6%)	37 (32.5%)
Places of residence [*N* (%)]			
City	84 (65.6%)	68 (61.3%)	86 (75.4%)
Town/Country	41 (32.0%)	41 (36.9%)	22 (19.3%)
Unknown	3 (2.3%)	2 (1.8%)	6 (5.3%)
Paternal education [*N* (%)]			
≥University	61 (47.7%)	34 (30.6%)	56 (49.1%)
Junior college	29 (22.7%)	20 (18.0%)	20 (17.5%)
High school	24 (18.8%)	18 (16.2%)	18 (15.8%)
≤Junior school	10 (7.8%)	36 (32.4%)	14 (12.3%)
Unknown	4 (3.1%)	3 (2.7%)	6 (5.3%)
Maternal education [*N* (%)]			
≥University	63 (49.2%)	25 (22.5%)	59 (51.8%)
Junior college	34 (26.6%)	25 (22.5%)	20 (17.5%)
High school	17 (13.3%)	29 (26.1%)	17 (14.9%)
≤Junior school	11 (8.6%)	29 (26.1%)	12 (10.5%)
Unknown	3 (2.3%)	3 (2.7%)	6 (5.3%)
Siblings [*N* (%)]			
None	61 (47.7%)	48 (43.2%)	53 (46.5%)
One	61 (47.7%)	57 (51.4%)	54 (47.4%)
≥Two	3 (2.3%)	3 (2.7%)	1 (0.9%)
Unknown	3 (2.3%)	3 (2.7%)	6 (5.3%)

HFASD, high-functioning autism spectrum disorder; DSLDs, developmental speech and language disorders; TD, typically developing.

### Comparisons of intelligence profiles among the HFASD, DSLDs and TD groups

Intelligence profiles of the HFASD, DSLDs and TD groups were presented and compared in Table 2. Similarity, Vocabulary, Comprehension, Block Design, Picture Concept, Matrix Reasoning, Digit Span, Coding and Symbol Search had been performed for every participant. Given the fact that some participants had never studied English alphabet before, part of the subjects were tested for Letter-Number Sequencing and the others were tested for Arithmetic. Results showed that all the subtests of WISC in HFASD group and DSLDs group were significantly lower than those in TD group (*P *< 0.05). In addition, all the indices including VCI, PRI, WMI, PSI and FSIQ in HFASD group and DSLDs group were significantly lower than those in TD group (*P *< 0.01). Participants in HFASD group achieved the highest average score in Block Design and the lowest average score in Comprehension among all the subtests. Children with DSLDs obtained the highest average score in Matrix Reasoning and the lowest average score in Digit Span. Comprehension, Picture Concept, Coding and Symbol Search scores of HFASD group were remarkably lower than DSLDs group, whereas Similarity and Block Design scores of HFASD group were remarkably higher than DSLDs group (*P *< 0.01). PRI was the highest index score and PSI was the lowest index score for children with HFASD. PRI and PSI were the highest index scores and WMI was the lowest index score for children with DSLDs. Furthermore, PSI scores of HFASD group were significantly lower than those of DSLDs group (*P *< 0.01).

**Table 2:** Comparisons of intelligence profiles among the HFASD, DSLDs and TD groups.

WISC-IV	HFASD group (*n* = 128)	DSLDs group (*n* = 111)	TD group (*n* = 114)	Post hoc comparisons
Similarity	9.17 ± 4.28	7.41 ± 4.12	12.21 ± 2.68	HFASD < TD** DSLDs < TD** DSLDs < HFASD**
Vocabulary	7.91 ± 3.37	8.50 ± 3.10	10.89 ± 2.66	HFASD < TD** DSLDs < TD**
Comprehension	6.94 ± 3.33	8.69 ± 2.70	9.91 ± 2.72	HFASD < TD** DSLDs < TD** HFASD < DSLDs**
Block Design	11.80 ± 3.87	9.67 ± 3.46	12.74 ± 2.85	HFASD < TD* DSLDs < TD** DSLDs < HFASD**
Picture Concept	7.36 ± 3.18	8.50 ± 2.79	10.04 ± 2.73	HFASD < TD** DSLDs < TD** HFASD < DSLDs**
Matrix Reasoning	10.34 ± 3.05	10.04 ± 2.71	11.73 ± 2.57	HFASD < TD** DSLDs < TD**
Digit Span	7.97 ± 3.04	7.39 ± 2.59	9.89 ± 2.65	HFASD < TD** DSLDs < TD**
Letter-Number Sequencing	8.26 ± 3.00	7.46 ± 2.38	10.12 ± 2.66	HFASD < TD** DSLDs < TD**
Arithmetic	7.43 ± 2.94	7.42 ± 2.51	9.87 ± 2.80	HFASD < TD** DSLDs < TD**
Coding	7.54 ± 2.54	8.98 ± 2.76	10.38 ± 2.91	HFASD < TD** DSLDs < TD** HFASD < DSLDs**
Symbol Search	7.90 ± 3.02	9.21 ± 3.07	10.25 ± 2.93	HFASD < TD** DSLDs < TD* HFASD < DSLDs**
VCI	88.83 ± 17.01	89.77 ± 14.67	106.05 ± 12.28	HFASD < TD** DSLDs < TD**
PRI	98.61 ± 16.61	95.69 ± 15.19	109.20 ± 12.60	HFASD < TD** DSLDs < TD**
WMI	88.38 ± 14.56	85.19 ± 12.07	99.83 ± 12.60	HFASD < TD** DSLDs < TD**
PSI	86.72 ± 13.07	95.25 ± 13.19	101.89 ± 13.93	HFASD < TD** DSLDs < TD** HFASD < DSLDs**
FSIQ	88.82 ± 13.85	89.51 ± 12.98	105.99 ± 11.12	HFASD < TD** DSLDs < TD**

HFASD, high-functioning autism spectrum disorder; DSLDs, developmental speech and language disorders; TD, typically developing; WISC-IV, Wechsler Intelligence Scale for children-Fourth Edition; VCI, Verbal Comprehension Index; PRI, Perceptual Reasoning Index; WMI, Working Memory Index; PSI, Processing Speed Index; FSIQ, Full-Scale Intelligence Quotient.

** *P* < 0.01, *P < 0.05.

### Comparisons of adaptive behaviors among the HFASD, DSLDs and TD groups

Adaptive behaviors of the HFASD, DSLDs and TD groups were presented and compared in Table 3. All of them were evaluated for Communication, Functional Academics, Self-Direction, Social, Leisure, Community Use, Home Living, Health and Safety, and Self-Care scores. Results indicated that all the skill areas, GAC, Conceptual domain, Social domain and Practical domain in HFASD group were significantly lower than those in TD group (*P *< 0.01). Except for Self-Care scores, all the skill areas, GAC, Conceptual domain, Social domain and Practical domain in DSLDs group were remarkably lower than those in TD group (*P *< 0.05). Consistent with previous studies, Social domain was the weakest adaptive functioning area of HFASD people, which was significantly lower than children with DSLDs as well (*P *< 0.01). Social, Self-Direction, Communication, Leisure and Self-Care scores of HFASD group were remarkably lower than those in DSLDs group (*P *< 0.05). Additionally, for children with DSLDs, more difficulties had been found in Social and Self-Direction skill areas. Practical domain was a relative strength for individuals with HFASD and DSLDs.

**Table 3:** Comparisons of adaptive behaviors among the HFASD, DSLDs and TD groups.

ABAS-II	HFASD group (*n* = 125)	DSLDs group (*n* = 109)	TD group (*n* = 108)	Post hoc comparisons
Communication	7.90 ± 2.57	8.69 ± 3.14	11.05 ± 2.94	HFASD < TD** DSLDs < TD** HFASD < DSLDs*
Functional Academics	8.74 ± 2.73	8.21 ± 2.89	10.74 ± 2.86	HFASD < TD** DSLDs < TD**
Self-Direction	6.65 ± 2.18	7.27 ± 2.38	8.37 ± 2.57	HFASD < TD** DSLDs < TD** HFASD < DSLDs*
Social	5.90 ± 2.53	7.48 ± 3.05	9.24 ± 2.74	HFASD < TD** DSLDs < TD** HFASD < DSLDs**
Leisure	7.37 ± 2.72	8.20 ± 3.09	10.00 ± 2.57	HFASD < TD** DSLDs < TD** HFASD < DSLDs*
Community Use	8.74 ± 2.98	8.86 ± 2.95	10.70 ± 3.09	HFASD < TD** DSLDs < TD**
Home Living	8.04 ± 2.58	8.39 ± 2.99	9.33 ± 3.30	HFASD < TD** DSLDs < TD*
Health and Safety	8.58 ± 2.61	8.50 ± 2.98	10.24 ± 2.66	HFASD < TD** DSLDs < TD**
Self-Care	7.30 ± 2.72	8.17 ± 3.06	8.73 ± 2.66	HFASD < TD** HFASD < DSLDs*
GAC	85.76 ± 13.23	88.73 ± 15.31	98.74 ± 13.80	HFASD < TD** DSLDs < TD**
Conceptual domain	86.60 ± 12.21	88.23 ± 14.32	99.99 ± 13.79	HFASD < TD** DSLDs < TD**
Social domain	80.73 ± 14.15	87.41 ± 16.84	97.68 ± 13.69	HFASD < TD** DSLDs < TD** HFASD < DSLDs**
Practical domain	89.30 ± 13.42	91.02 ± 15.13	98.42 ± 14.74	HFASD < TD** DSLDs < TD**

HFASD, high-functioning autism spectrum disorder; DSLDs, developmental speech and language disorders; TD, typically developing; ABAS-II, Adaptive Behavior Assessment System-Second Edition; GAC, General Adaptive Composite.

** *P* < 0.01, *P < 0.05.

### Correlations between intelligence profiles and adaptive behaviors in the HFASD group and DSLDs group

Pearson correlations between intelligence profiles and adaptive behaviors in the HFASD group and DSLDs group were respectively presented in Tables 4, 5. Outcomes suggested that VCI, PRI, PSI and FSIQ scores of WISC were positively correlated with all the adaptive behavior domains including Conceptual domain, Social domain, Practical domain and GAC in HFASD group (*P *< 0.05). Moreover, WMI scores were significantly correlated with Conceptual domain scores for children with HFASD (*P *< 0.05). Conceptual domain had the strongest positive correlation with FSIQ in HFASD group (*r* = 0.473, *P *= 0.000), especially Functional Academics (*r* = 0.573, *P *= 0.000). For children with DSLDs, VCI, PRI, WMI and FSIQ scores were positively correlated with Conceptual domain scores (*P *< 0.05). WMI and FSIQ scores were positively correlated with GAC scores (*P *< 0.05). However, intelligence had no significant correlations with Social domain or Practical domain in DSLDs group. Compared with DSLDs group, IQs showed stronger relationships with adaptive behaviors in HFASD group.

**Table 4:** Pearson correlations between intelligence profiles and adaptive behaviors in the HFASD group.

ABAS-II		VCI	PRI	WMI	PSI	FSIQ
GAC	*r*	0.325	0.303	0.161	0.269	0.384
	*P*	0.000	0.001	0.073	0.003	0.000
Conceptual domain	*r*	0.402	0.377	0.228	0.291	0.473
	*P*	0.000	0.000	0.011	0.001	0.000
Social domain	*r*	0.338	0.277	0.135	0.299	0.382
	*P*	0.000	0.002	0.134	0.001	0.000
Practical domain	*r*	0.243	0.247	0.108	0.223	0.299
	*P*	0.007	0.005	0.229	0.013	0.001

HFASD, high-functioning autism spectrum disorder; GAC, General Adaptive Composite; VCI, Verbal Comprehension Index; PRI, Perceptual Reasoning Index; WMI, Working Memory Index; PSI, Processing Speed Index; FSIQ, Full-Scale Intelligence Quotient.

**Table 5:** Pearson correlations between intelligence profiles and adaptive behaviors in the DSLDs group.

ABAS-II		VCI	PRI	WMI	PSI	FSIQ
GAC	*r*	0.146	0.093	0.232	0.130	0.198
	*P*	0.132	0.334	0.015	0.179	0.039
Conceptual domain	*r*	0.275	0.225	0.326	0.152	0.332
	*P*	0.004	0.019	0.001	0.117	0.000
Social domain	*r*	0.151	0.102	0.150	0.142	0.186
	*P*	0.118	0.289	0.119	0.142	0.052
Practical domain	*r*	0.042	−0.022	0.178	0.101	0.087
	*P*	0.663	0.820	0.065	0.298	0.367

DSLDs, developmental speech and language disorders; GAC, General Adaptive Composite; VCI, Verbal Comprehension Index; PRI, Perceptual Reasoning Index; WMI, Working Memory Index; PSI, Processing Speed Index; FSIQ, Full-Scale Intelligence Quotient.

## Discussion

HFASD and DSLDs are common neurodevelopmental disorders with molecular biological evidence and imaging basis. Existing research has discovered genetic defects and brain morphological alterations or structural abnormalities in HFASD and DSLDs patients (27–32). The aforementioned pathophysiology mechanisms result in distinctive clinical symptoms, cognitive abilities and adaptive performances of HFASD and DSLDs. Previous studies investigated the intelligence profiles and adaptive functioning characteristics of them, but few studies compared the similarities and differences between them. Our study made comparisons of intelligence levels and adaptive behaviors and explored their interactions in the HFASD and DSLDs groups, which further provided clinical evidence for differential diagnosis and prognosis of HFASD and DSLDs. It was of great importance to expand insights into clinical features of the two neurodevelopmental disorders.

In line with previous research findings (4, 5, 7, 33), the present study convinced PRI was the strongest index and PSI was the weakest index in WISC for children with HFASD. Our results showed participants in HFASD group achieved the highest average score in Block Design and the lowest average score in Comprehension among all the subtests. Coding and Symbol Search in HFASD group were significantly lower than those in DSLDs group and TD group. The largest discrepancies between HFASD and TD group existed in VCI and PSI scores. Above findings reflected difficulties mainly in processing speed and verbal comprehension and advantages in perceptual reasoning for children with HFASD. These phenomenon displayed the impairments of children with HFASD in understanding social rules and sensory processing (5). They also connected phenotypes of HFASD with neuroimaging structures. It had been discovered that increased amygdala size correlated with the severity of social and communication deficits in ASD (29). Thus, our research provided more sufficient evidence supporting the conclusions from previous studies. Consistent with partial previous outcomes (5, 6), the average FSIQ of HFASD group was significantly lower than that of TD group in our study. However, several research held the view that FSIQ of HFASD children had no significant difference with TD children (4, 7). The discrepancy might be caused by limited sample size. Additionally, HFASD children had weaknesses in auditory attention and graphomotor skills. Although WISC-IV captured HFASD children's visual reasoning strength with non-verbal Matrix Reasoning and Picture Concept subtests, it might still underestimate the intelligence of HFASD to some extent as a measurement. Therefore, more large population-based studies with appropriate measurements are required to elucidate this issue in the future.

One noteworthy finding from this study was that IQ scores of all the subtests and indices in WISC of DSLDs children were significantly lower than TD children and WMI was the most severe impaired index for DSLDs individuals. As far as we are concerned, few previous studies systemically explored the intelligence profile of children with DSLDs (34, 35). Our study comprehensively displayed the cognitive characteristics of DSLDs, suggesting DSLDs affect multiple learning abilities at childhood and adolescence. Results demonstrated that children with DSLDs obtained relative lower scores in Similarity, Digit Span, Arithmetic and Letter-Number Sequencing. But they achieved relative higher scores in Matrix Reasoning, Block Design and Symbol Search. Children with DSLDs showed a good competence in perceptual reasoning and processing speed. However, poor performance in VCI and WMI indicated their predominant defects in verbal comprehension and executive functions. Executive attentional control in working memory was implied to be a powerful predictor of language development and a strong determinant of learning capability (regarding both literacy and numeracy) (35–38). Our findings further confirmed children had a history of DSLDs were at increased risks of adverse long-term consequences on academic achievements.

Another interesting finding of our study was that VCI, PRI, PSI and FSIQ scores of WISC were positively correlated with all the adaptive behavior domains including Conceptual domain, Social domain, Practical domain and GAC in HFASD group. Research of Lopata et al. (17) and Tamm et al. (18) reported significant discrepancies between IQ scores and adaptive functioning scores for children with HFASD. Kraper et al. (39) observed a similar phenomenon in young adulthood patients of ASD. Different from previous studies, results of our study displayed smaller gaps between cognitive levels and adaptive behaviors. There existed several factors influencing the outcomes. Unbalanced parental educational backgrounds of participants was speculated to be the reason of lower IQ scores in our HFASD group compared with literature reports. It should also be taken into consideration that overprotection from parents or grandparents and lack of exercise in daily life were important factors interfering progress in adaptive behaviors for Chinese children. Additionally, we found significant positive correlations between IQs and adaptive behavior scores for children with HFASD, which was a novel contribution of our research. Kenworthy et al. (20) and Audras-Torrent et al. (40) once discovered IQ scores were positively associated with adaptive communication skills and Conceptual domain. Rosa et al. (41) reported worse intellectual performance, especially in verbal comprehension and working memory, was significantly correlated with more severe symptoms and poorer adaptive functions. Our results revealed IQ scores (VCI, PRI, PSI and FSIQ) of HFASD were positively correlated with GAC and all the domains (Conceptual domain, Social domain and Practical domain) of ABAS-II. The strong associations suggested intelligence level was a potentially important factor that affected the development of adaptive behaviors for children with HFASD, which was contributed to predicting clinical outcomes (i.e., work performance, self-care, friendship, prosocial behavior and community integration in the adolescence and adulthood) of HFASD patients.

Unlike children with HFASD, cognitive capabilities exerted less impacts on adaptive behaviors in children with DSLDs. Our outcomes convinced significantly poorer adaptive functions of all the domains in children with DSLDs compared with TD children. We also found VCI, PRI, WMI and FSIQ scores of DSLDs group were positively correlated with Conceptual domain. But their associations with Social domain and Practical domain were not statistically significant. WMI and FSIQ scores of DSLDs group were positively related with GAC. The relationships between IQs and adaptive behaviors in DSLDs group were not as strong as counterparts in HFASD group. Moreover, children with DSLDs had balanced scores of all the domains in ABAS-II. Nevertheless, children with HFASD demonstrated striking impairments in Social domain. This discovery proved the underlying biological mechanisms of ASD. It had been reported that functional abnormalities in orbitofrontal-striata-amygdala circuit led to difficulties in social orienting, social seeking and liking, and social maintaining, which resulted in social impairments in ASD children (42). Our findings further disclosed the similarities and differences in cognitive capabilities and adaptive behaviors of the two neurodevelopmental disorders.

There were still some limitations in the present study. First of all, our sample lacked population diversity and the sample size was not big enough. Given that China is a developing country characterized by huge population and imbalanced economic development, a multicenter study with larger sample size and multi-nationalities can be more representative. Secondly, the ages and genders of our participants were not well matched. Median age of TD group was greater than those of HFASD and DSLDs groups. Due to the characteristics of diseases, amounts of female participants in the HFASD and DSLDs groups were limited. Better matched research is required for minimizing the influences by confounders such as age and gender. Thirdly, data of ABAS-II were self-reported and possibly be subject to recall bias. Finally, we cannot rule out the possibility of underestimating intelligence levels for children with HFASD. As we all know, IQ scores of WISC highly depend on the cooperation degree and oral verbal comprehension of subjects. Whether WISC an appropriate measurement evaluating cognition of HFASD is still a controversy and worths further investigation in the future. Therefore, more well-designed prospective studies with innovative methods are needed to clarify this issue.

## Conclusion

Our study convinced the consequences of previous studies regarding intelligence profiles and adaptive behaviors in children with HFASD and DSLDs. Both HFASD and DSLDs showed impairments in verbal comprehension and executive functions. Processing speed and working memory were the predominant defects of children with HFASD and DSLDs in the field of executive functions, respectively. Perceptual reasoning was a relative strength for them. Children with DSLDs had balanced scores of all aspects in adaptive behaviors; whereas HFASD individuals demonstrated striking impairments in Social domain. Moreover, this study made breakthroughs in discovering positive correlations between IQs and adaptive functions in children with HFASD and DSLDs. Compared with DSLDs, intelligence displayed stronger relationships with adaptive behaviors in HFASD. Our research findings contributed to more profound insights of the two neurodevelopmental disorders.

## Data Availability

The raw data supporting the conclusions of this article will be made available by the authors, without undue reservation.
